# Inconsistencies in dosage practice in children with overweight or obesity: A retrospective cohort study

**DOI:** 10.1002/prp2.398

**Published:** 2018-04-20

**Authors:** Christina Gade, Hanne R. Christensen, Kim P. Dalhoff, Jens Christian Holm, Helle Holst

**Affiliations:** ^1^ Department of Clinical Pharmacology Copenhagen University Hospital Bispebjerg and Frederiksberg Copenhagen NV Denmark; ^2^ Children's Obesity Clinic European Center of Management (EASO) Department of Paediatrics Zealand University Hospital Holbaek Denmark

**Keywords:** childhood obesity, clinical pharmacology, dosing regimens, overweight, pediatrics

## Abstract

Obesity can affect the pharmacokinetics of most drugs, which may result in under‐ or overdosing if traditional pediatric dosing strategies are used. To investigate currently applied dosage strategies in children with overweight or obesity (overweight/obesity), in a clinical treatment facility. In particular, whether dosing guidelines were available and metrics of body size applied. A retrospective cohort study of 200 patients admitted to the Danish Children′s Obesity Clinic. Data were collected from 2007 to 2015. Overweight/obese children 3‐18 years were included if they had at least one drug prescription. Overall there were 658 prescriptions, primarily analgesics, psychotropics, asthma medications, and antibiotics. Except for one prescription, guidelines for dosage of overweight/obese children were not available in the clinic. In one prescription of gentamicin, the dose was adjusted by a metric body size. Otherwise dose was predominately prescribed either by total body weight or as fixed dose by age, in accordance with the recommendations of normal weight children. In drugs with a narrow therapeutic interval, we found large interindividual variations in dosing regimens, that is, for gentamicin, paracetamol, and prednisolone. Reduction of dose to the maximum recommended adult dose was common practice, when the dose calculated by total body weight (ie, mg/kg) exceeded this maximum. This study highlights the shortage of dosing guidelines in overweight/obese children. We found a large interindividual variability in dosage regimens, even in drugs with narrow therapeutic intervals. The clinicians rely on “best practice”, as evidence‐based dosage regimens are missing for many drugs prescribed during childhood.

**Abbreviations**
ABW: adjusted body weight
ATC: anatomical therapeutic chemical
BMI: body mass index
CL: clearance
IBW: ideal body weight
LBW: lean body weight

## 1 INTRODUCTION

Obesity is associated with a wide range of serious health complications, including asthma, musculoskeletal disorders, depression, sleep apnea, dyslipidemia, hypertension, steatosis, diabetes type 2, and numerous cancers.^1^, ^2^ Consequently, children who are overweight and obese are more likely to receive drug treatment than their normal weight peers.^3^, ^4^

### 1.1 Drug dosing in normal weight children and challenges in overweight/obese children

One of the most common methods for drug dosing in children in pediatric clinical practice is to extrapolate adult dose by body weight, eg mg per kilogram (kg).^5^ This approach represents an a priori assumption of a linear relationship between weight and dose, as dose doubles with a twofold increase in the weight of a child.^5^ Another common method for dosing in children is based on age, by dividing the pediatric population into subcategories of age. This method introduces an artificial discontinuity in the dose‐response relationship across each age category.^5^As a result of the high weight relative to height in children with overweight/obese, a linear dosing by total body weight (TBW) might even exceed the maximum recommended dose in adults, while fixed dosage by age may result in subtherapeutic doses.^5^ In addition, the principal pharmacokinetic (PK) determinants of drug dosing, that is, volume of distribution (*V*_d_) and clearance (CL) are both often altered in obese patients. This is partly due to changes in physiology and body composition, with a relatively higher increase in fat (60%) compared to lean tissue (40%) per kg of TBW, as well as lean mass is more hydrated, which is attributed to increased extracellular water.^6^ In addition, alterations in drug binding proteins, cardiac output, organ blood flows, and tissue perfusion may influence the PK.^7^, ^8^ Even though, the hepatic metabolism and the influence of obesity in children not has been studied extensively.^8^ Further, PK data in this subgroup is not required for approval of new drugs by default by the Danish health authorities or the European Medicine Agency (EMA). The lack of clinical trials in children, who are overweight/obese therefore prompts clinicians to extrapolate dosing strategies from studies in adults with overweight. However, such extrapolation strategies ignore differences in drug disposition (distribution and elimination) characteristics, reflecting children's general degree of immature metabolic pathways and physiological development. In addition, pathophysiological alterations accompanying obesity are assumed to have a similar influence on PK parameters in children, who are overweight/obese as in adults.^5^, ^6^, ^9^, ^10^ Ideal body weight (IBW), lean body weight (LBW), adjusted body weight (ABW) and allometric scaling, see Table 1. Whether the metrics are actually used in clinical practice is uncertain.

**Table 1:** Metrics of body size, used to calculate dose in children with overweight/obese

Metrics of body size	Formula
TBW, kg	Total body weight
LBW, kg	Male: (1.1 × TBW)–(0.0128 × BMI × TBW) Female: (1.07 × TBW)–(0.0148 × BMI × TBW)
IBW, kg	BMI 50th percentile for age and gender (children)
ABW, kg	IBW + (0.4 × (TBW–IBW)
BSA, m^2^	TBW × 0.024265 × height (cm)0.3964 × TBW (kg) 0.5378 or (TBW × height (cm)/3.600)½
Allometric scaling	CL _child_ = CL_adult_(TBW_child_/70)b b = the allometric coefficient for CL, usually set at 0.75. Oral dose in steady state (ss) is then: Dose_oral_ = C_av,ss_ × CL × Ϯ/F

LBW, lean body weight; ABW, adjusted body weight; BMI, body mass index; IBW, ideal body weight; BSA, body surface area; *C*_av_, average plasma concentration in steady state; Ϯ, dose interval; *F*, bioavailability.

### 1.2 Objectives

To investigate currently applied dosing strategies in children, who are overweight/obese, in a clinical treatment facility. In particular, whether dosing guidelines were available and metrics of body size applied.

## 2 MATERIALS AND METHODS

### 2.1 Design and setting

A retrospective cohort study was conducted at the Children′s Obesity Clinic, Department of Paediatrics, Holbaek University Hospital in Region Zealand, in the period 2007‐2015. The study was approved by the Data Protection Agency (BBH‐2014‐045, I‐suite 03045).

### 2.2 Study participants

Two hundred consecutively enrolled overweight/obese children aged 3‐18 years, entering the chronic care multidisciplinary intervention program at the Children′s Obesity Clinic. Only children treated with at least one drug were included. Children had been referred from their general practitioners (GPs), school‐ and community‐based doctors, or pediatricians. Exclusion criteria were a Body Mass Index (BMI) Standard Deviation Score (SDS) ≤ 1.28, which corresponds to the 90th percentile according to the Danish age and sex‐adjusted references.^11^

### 2.3 Data collection

Data collected comprised patient demographics including: date of birth, gender, comorbidity, height, weight, and all drug prescriptions from Department of Paediatrics or Division of Child and Adolescent Psychiatry at entering day and during the follow up period. Data collection was performed by a medical doctor from Department of Clinical Pharmacology, Copenhagen.

### 2.4 Outcomes and measures

The following data were collected for each specific drug: Indication for treatment according to the International Classification of Diseases version 10 (ICD‐10),^12^ drug dose (milligram), dosage interval, duration of treatment, age of child at prescription time (years), weight (kg), and height (cm). For evaluating dosage strategies, the following data were registered: dosage by total body weight (TBW), fixed dose by age (years), use of adjusted weight measures (eg LBW, BSA, IBW, ABW) or dose estimation by other strategies. If available, specific drug dosing guidelines for overweight/obese children would be used as reference document; Guidelines for each drug prescribed were searched in the local pediatric treatment facility, herein the electronic prescription system; “Opus Medicine”. Furthermore, at the official Danish Websites: Pro.medicin.dk, The Danish Society of Paediatricians′ http://www.paediatri.dk, and the Summary of Product Characteristics (SmPC).

For standardization, the WHO Anatomical Therapeutic Chemical (ATC) Classification System for medication was used. WHO‐ATC uses a hierarchical system for classifying medicines into distinct groups at five levels according to the organ or system on which they act and their chemical, pharmacological and therapeutic properties. Dosing strategy was determined by simple backwards calculation of total dose (eg in mg per kg) and with concordance to the available dosing guidelines. Dosing strategies found were discussed continuously with a group of senior MDs in Clinical Pharmacology and—Pediatrics.

### 2.5 Statistical analysis

Continuous variables are presented as number of observations (percentages) or median (range), while categorical (nominal) variables are presented, using number of observations or frequencies as percentages. Data management was conducted, using R, version 3.2.2. The age of the child at first visit was stratified into two groups: 2‐11, 12‐18 years according to the International Conference on Harmonization's, ICH‐11 criteria.^13^

## 3 RESULTS

Demographic characteristics are summarized in Table 2. In total, 1372 patient records and medication charts were reviewed in order to achieve the predefined sample of 200 patient records comprising at least 1 prescription in the study period. The cohort included 63% females, almost equally divided into the two subpopulations of 3‐11 years (48%) and 12‐18 years (52%), respectively. In males, the 3‐11 years old constitute the largest group (62%).

**Table 2:** Demographic characteristics at the first visit to The clinic of obese children

Characteristics	Total	2‐11 years	12‐18 years
N (%)	200	107 (53.5)	93 (46.5)
Age, years (median, range)	11 (3‐18)	10 (3‐11)	14, 5 (12‐18)
Male (%)	126 (63)	60 (48)	66 (52)
Female (%)	74 (37)	46 (62)	28 (38)
BMI *z‐*score (median, range)	2.95 (1.28‐9.72)	2.98 (1.64‐9.72)	2.92 (1.28‐7.78)
Distribution of co‐morbidities, ICD‐10‐code and diagnosis: (%)	105 (100)^a^	56 (53)	49 (47)
D44.4‐D69.3 Craniophanygioma, Autoimmune hemolytic anemia	3	1	2
E03.9‐E.70 Myxoedama, Autoimmune thyroiditis, Type 1 diabetes, Type 2 Diabetes Mellitus, Polycystic ovarian syndrome, Precocious puberty, Endocrine disorder, unspecified, Disorders of aromatic amino‐acid metabolism	12	5	7
F32‐F99.2 Depressive episode, Other anxiety disorders, Borderline personality, Infantile autism, Asperger syndrome, Attention‐deficit hyperactive disorder, Tourette disorder	19	6	13
G40 Epilepsy	4	3	1
I10 Hypertension	2	1	1
J45 Asthma	43	30	13
K21‐59 Gastroesophageal reflux disease, Constipation	7	3	4
L20‐L40 Atopic dermatitis, Psoriasis	4	2	2
M00‐M99 Musculoskeletal disorders, Low back pain	3	1	2
Q14‐Q89.3 Congenital malformations of posterior segment of eye, Other congenital malformations of male genital organs, Other congenital musculoskeletal deformities, Bardet‐Biedl syndrome, Dextrocardia with situs in versus	5	3	2
R32 Unspecified urinary incontinence	1	0	1
T78.4 Allergy, unspecified	2	1	1

ICD, international classification of diseases.

Data are presented as median (range) or frequencies in numbers or percent.

a Some of the patients are represented in more than one group.

**Figure 1:**
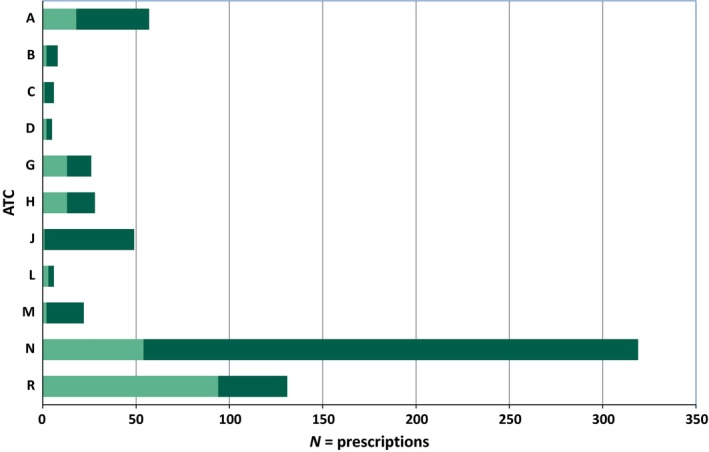
Distribution of prescriptions registered at first visit and distribution of prescriptions conducted during hospitalization. In total: N = 658 prescriptions. Light green color bar chart: 203 prescriptions registered at the first visit. Dark green color bar: 455 prescriptions registered during hospitalization. Some of the children are represented in more than one ATC group

Distribution of all drug prescriptions in accordance to the ATC classification system is illustrated in Figure 1. Totally 658 prescriptions were registered, of these were 203 registered at the first visit. A majority of the prescriptions (46%) were respiratory drugs (ATC group R), followed by drugs acting on the nervous system (ATC group N; 27%) , for example, central nervous system stimulants and analgesics, and drugs acting on the alimentary tract and metabolism (ATC group A; 9%), mainly laxative drugs and proton pump inhibitors. The remaining 18% were distributed between various groups. In the follow‐up period, during hospitalization, a total number of 455 prescriptions were registered, primarily within the ATC groups as registered at the primary visit (groups N, A & R), in addition to antibiotics (ATC group J). In accordance with these prescription patterns, the majority of comorbidities comprised of 41% (43/105) asthma diagnoses (ICD‐10 code J45), while 22% had a mental—and behavioral disorder (codes F32 to F99.2) or disorders of the nervous system (code G40),Table 2. Eleven percent had an ICD‐code of endocrine, nutritional and/or metabolic disorder (codes E03.9 to E.70) and 7% had a disorder of the digestive system (codes K21 to K59). The remaining 19% was widely distributed between various comorbidities.

### 3.1 Dosing strategies

Distribution of the individual drugs prescribed at ATC‐level five, and the dose regimen used in the follow up period is presented in Table 3. Drugs were prescribes approximately fifty‐fifty as fixed dose by age (N = 183) or by TBW (N = 179), and in accordance with the SmPCs.

**Table 3:** Distributionen on individual drugs at ATC‐level five and the dose regimen used. Each patient can be represented in more than one group

Drug (ATC‐code, Genericum)	Number of prescriptions N:455	Fixed dose by age N:183	Dosed per total kilogram body weight N: 179	Dose estimation N:28	Age (median, range)
A10BA02, insuline detemir	1	—	—	—	10
A02AA04, magnesium oxide	1	—	—	—	16
A02BC01, omeprazole	4	4	—	—	13 (10‐16)
A02BC02, pantoprazol	3	3	—	—	17 (13‐18)
A02BC03, lansoprazole	4	3	—	—	17 (13‐18)
A02BC05, esomeprazole	1	1	—	—	18
A02BX13,gaviscon^®^	1	1	—	—	16
A06AB08,sodium picosulfate	2	2	—	—	15.5 (15‐16)
A06AD11, lactulose	1	1	—	—	10
A06AD65, Movicol^®^	12	12	—	—	12 (4‐16)
A07EC02, mesalazine	4	4	—	—	16 (13‐16)
A08AB01, orlistat	2	—	—	—	13
A10BA02, metformin	1	1	—	—	17
A10AB05, aspartat^1^	1	—	—	—	10
A11CC05, cholecalciferol	1	1	—	—	16
B01AA03, warfarin	1	1	—	1	18
B01AB04, dalteparin	1	—	1	1	18
B02AA02, tranexamic acid	1	—	1	—	17
B03BB01, folic acid	1	—	—	1	14
B03AA02,ferrous fumerat/vitamin C^2^	1	—	—	—	13
B03AA07,ferrous sulfate	1	—	—	—	16
C07AA05, propranolol	1	1	—	1	15
C03CA01, furosemide	1	—	1	1	13
C08CA01, amlodipine	1	1	—	—	13
C09AA01, captopril	2	—	1	1	16
D07AB02, hydrocortisone butyrate	1	—	—	—	10
D07AD01, clobetasolpropionat	1	1	—	—	11
D10AE51, benzoyl peroxide, clindamycin	1	1	—	—	13
G03AA07, COC^3^, 2.generation progestin	7	—	—	—	17 (15‐17)
G03AC01, norethisteron, 1.generation	1	—	—	—	17
G03AC09, desogestrel, 3.generation	2	—	—	—	17 (16‐18)
G03DA02, medroxyprogesteronacetate	1	—	1	—	15
G04BD08, solifenacin	2	—	—	—	15.5 (11‐16)
H01BA02, desmopressin	1	1	—	—	8
H02AB02, dexamethason	1	—	—	—	12
H02AB04, methylprednisolone	3	—	3	2	16 (15‐16)
J01CA01, ampicillin	3	—	3	—	6 (5‐17)
J01CA08, pivmecillinam	4	—	4	2	13 (5‐15)
H02AB06, prednisolone	10	2	3	2	17 (10‐18)
J01CA04, amoxicillin	2	2	—	—	5
J01DC02, cefuroxime	2	1	1	—	16 (14‐18)
J01CE01, benzylpenicillin	3	—	3	—	11 (10‐17)
J01CE02, phenoxymethylpenicillin	8	—	1	—	13.5 (6‐17)
J01CF01, dicloxacillin	5	—	3	—	11 (11‐13)
J01DH02, meropenem	1	1	—	—	16
J01EE01, sulfametoxazol/trimetoprim	1	1	—	—	17
J01FA09, chlarithromycin	2	—	—	—	18
J01FA10, azithromycin	4	4	—	—	16.5 (11‐18)
J01GB03, gentamicin	6	—	5	2	5 (5‐18)
J01MA02,ciprofloxacin	4	2	2	1	16.5 (16‐18)
J01XE01, nitrofurantoin	2	1	—	—	11.5 (7‐16)
J05AB01, acyclovir	1	—	—	—	11
L02AE02, leoprorelin	2	—	—	—	13
L04AB02, Infliximab	1	—	—	—	16
M01AE01, ibuprofen	11	11	—	—	13 (11‐18)
M01AE02, naproxen	1	1	—	—	18
M01AB05, diclofenac	7	—	—	—	15 (6‐17)
M03BB03, chlorzoxazone	1	1	—	—	18
N02AA01, morphine	7	1	6		14 (13‐18)
N02AX02, tramadol	2	2	—	—	17, 5 (17‐18)
N02BE01, paracetamol	34	2	15	9	13 (5‐18)
N05AF03, chlorprothixen	12	1	—	—	14 (13‐18)
N05AH03, olanzapine	1	—	—	—	14
N05AH04, quetiapine	15	—	—	—	14 (10‐18)
N05AX08, risperidone	2	2	—	—	10
N03AX09, lamotrigene	4	4	—	—	14
N05AX12, aripiprazole	15	4	—	—	13.5 (10‐16)
N05BA01, diazepam	2	2	—	—	12 (11‐13)
N05BA04, oxazepam	1	1	—	—	18
N05CF01, zopiclone	1	—	—	—	16
N05CH01, melatonin	12	—	—	—	13.5 (8‐15)
N06AB03, fluoxetine	6	6	—	—	15.5 (11‐16)
N06AB04, citalopram	6	1	—	—	11 (10‐18)
N06AB06, sertraline	15	5	—	—	15 (11‐16)
N06AX11, mirtazapine	2	—	—	—	17
N06AX16, venlafaxine	1	—	—	—	17
N06BA04, methylphenidate	109	43	62	4	11 (4‐17)
N06BA09, atomoxetine	12	—	12	‐	13.5 (10‐17)
N06BA12, lisdexamfetamine	6	6	—	—	16 (15‐16)
R03DC03, montelukast	4	4	—	—	11.5 (6‐15)
R06AD02, promethazine	3	3	—	—	13 (11‐17)
R03AC02, salbutamol	7	7	—	—	13 (6‐17)
R03AC03, terbutaline	7	7	—	—	12 (9‐15)
R03BA02, budesonide	8	8	—	—	11 (9‐18)
R03BA05, fluticasone propionate	3	3	—	—	10 (6‐12)
R01AD12, fluticasone furoate	2	2	—	—	12.5 (11‐14)
R06AA04, clemastine	1	1	—	—	16
R06AE07, cetirizine	2	2	—	—	13.5 (10‐17)
V03AB23, acetylcysteine	1	—	1	—	16

Off label use (Indication, age, dose, form and dose interval) has not been specified in the table. Some drugs are not dosed by weight or age, that is, insulin detemir, which is dosed by blood glucose level. For drugs prescribed off label or off license, it was not possible to specify dose regimen. Dose estimation comprises dose capping and use of metrics of body size. The later only registered in one case, which was gentamicin dosed by ABW, with reference to the Danish website http://www.promedicin.dk.

Specific guidelines for dosing strategies in children with overweight/obese were not available for any of the drugs prescribed. Neither at the Children's obesity clinic, or at the official website of The Danish Society of Paediatricians, which contains various national pediatric dosing guidelines,^14^ nor were there any supportive dosing parameters incorporated in the electronic system; “Opus Medicine”. Further, The Danish Website: pro.medicin.dk^15^ only provides prescription guidelines for gentamicin for obese adults. The metric used for body size is adjusted by weight (ABW).

*In the ATC group J (antibiotics)*, gentamicin was prescribed in 4 of 200 patients, which comprised in total 6 prescriptions. They were dosed after three different dosage regimens, either by TBW, ABW, or dose‐capping. Toxicity was not reported and Therapeutic Drug Monitoring (TDM) was performed in all cases. Phenoxy‐methyl penicillin was prescribed as a fixed dose in accordance with adult regimen in the majority of the children, as a dose of 1.000.000 IE three times per day. In only one patient, the dose was calculated by TBW in accordance to the SmPC, whereas both Benzyl‐penicillin (i.v) and Ampicillin (i.v.) were dosed by TBW, Table 2.

*Systemic use of prednisolone (ATC group H)* were registered in totally 10 cases, prescribed by various doses depending on indication, which were allergic reaction, angioedema, asthma and autoimmune hemolytic anemia, median dose 25 mg/day (range 2.5‐50 mg/day). Methylprednisolone was prescribed in three cases also with dose primarily depending on indications, which were asthma, allergic reaction, and colitis ulcerosa, median dose was 40 mg/day (range 40‐80 mg/day).

*For the ATC group M*, which comprises nonsteroidal anti‐inflammatory drugs, ibuprofen was prescribed as a fixed dose of 400 mg three times/day in the majority of prescriptions, whereas diclofenac was dosed as 50 mg three time per day in 5 of 7 prescriptions and as 150 mg three times per day, and at last as 100 mg as a single dose.

*For ATC group N:* In total, 34 prescriptions of paracetamol were registered. In 15 cases, paracetamol was dosed by TBW in accordance with a normal weight children dosing regimen and in 9 cases dose‐capping was registered when dose exceeded the recommended maximum adult dose and in 3 cases dose prescribed exceeded the dose calculated by TBW. In one case, the recommended maximum adult dose was exceeded. In total, 7 prescriptions of morphine were registered, of these 5 were prescribed as subcutaneous administrations, dosed by TBW in accordance to the SmPC, and 2 prescriptions were administered as tablets, both as 5 mg per dose, which in both cases was lower than the recommended initial dose for normal weight children. Only 2 prescription of diazepam was registered as a dose of 10 mg, which is the maximum recommended dose for treatment of seizures in children in general, and in accordance to the recommendations at The Danish Society of Paediatricians official website.^14^ Further, 4 prescriptions of lamotrigine were registered all prescribed as fixed dose by age during maintenance treatment of epilepsy.

*For ATC group R:* 32 registrations of asthma medications were registered, herein inhaled beta‐2 agonists, inhaled corticosteroids, fixed dose combination, and montelukast. All drugs in group R were prescribed as fixed dose by age.

## 4 DISCUSSION

This study gives an overview of clinical practice in dosing of overweight/obese children in different pediatric settings. We found no specific dosing guidelines available for overweight/obese children in the investigated clinics for any of the recorded drugs. As limited data are available in overweight/obese children for most drugs, the results of this study will most likely apply to other pediatric settings in Denmark as well as internationally.

### 4.1 Antibiotics

In line with other studies,^16^, ^17^ we found a noticeable prescription of antibiotics (ATC group J), of which, it is well known that in addition to therapeutic failure, inappropriate dosage can lead to antibiotic resistance, and drug‐related toxicity. Nonetheless, we found only one overweight/obese child dosed by an adjusted metric of body size, which was gentamicin dosed by ABW in treatment of pyelonephritis. It is generally accepted that the initial empiric dose (loading‐dose) is critical to achieve an early high peak concentration of gentamicin that directly correlates with maximal killing of the pathogen.^18^ One PK study of gentamicin has been performed in overweight/obese children,^18^ and based on the results, it has been suggested that the total daily dose per kilogram bodyweight should be based on ABW, similar to adults.^18^, ^19^ Nonetheless, we found three other overweight/obese children, who had gentamicin prescribed by other and different dosing strategies. Herein, a 18‐year‐old female with a weight of 106 kg, height 169.3 cm, who received a loading dose of 240 mg gentamicin prescribed in the treatment of pneumonia. The dose prescribed was 39% (152 mg) lower than the dose calculated by ABW (392 mg). Opposite, a 5‐year‐old female with a weight of 29.6 kg, height 106.4 cm, received a loading dose of 180 mg, dosed by TBW (6 mg kg^−1^). The dose prescribed was 60% (67.5 mg) higher than the ABW‐based dose of 112.5 mg.

Despite their widespread use, there is very little information regarding dosing penicillin in obese patients.^20^ We found phenoxy‐methyl penicillin prescribed as a fixed dose in accordance with adult regimen in 7 out of 8 children. Consequently, a 6‐year‐old child had an approximately fourfold higher daily dose per kg body weight, than the 17‐year adolescent (88 mg kg^−1^ day^−1^ vs 21 mg kg^−1^ day^−1^). For penicillin's in which time of the drug concentration above the minimum inhibitory concentration (T > MIC) is important, it follows that increasing doses or frequency will improve the PD. Furthermore, the therapeutic index for penicillin's is wide, and it therefore seems reasonable to dose penicillin by TBW in overweight/obese children. However, this has to be investigated further, and PKPD studies of penicillin in children, who are overweight/obese are therefore in demand.^16^, ^20^

### 4.2 Analgesics

We found a large interindividual dosing of paracetamol, and the capping dose was common practice. In a recent study by Rongen et al.,^21^ the pharmacokinetics of paracetamol in morbidly obese adult patients was investigated, with a specific emphasis on Cytochrome (CYP) 2E1 mediated CL. Paracetamol plasma concentrations were significantly lower in obese patients. Thus, an increased dose of paracetamol may be anticipated to achieve a better pharmacodynamic response in obese patients. However, the induced CYP2E1‐activity may also worsen the safety profile of paracetamol due to higher concentrations of the toxic metabolite N‐acetyl‐p‐benzoquinone (NAPQI). As paracetamol is the most prescribed drug in pediatric wards internationally,^22^ PKPD studies in overweight/obese children is of outmost importance to clarify this question.

#### 4.2.1 Ibuprofen

We found ibuprofen primarily prescribed as a fixed dose of 400 mg. Reduced peak, increased *V*_d_ has been found for ibuprofen in adults with obesity,^23^ indicating dose may have to be increased. Ibuprofen has not been investigated in children overweight/obese. Due to common severe adverse effects such as gastrointestinal bleeding; increasing dose by extrapolation of adult data cannot be recommended as standard in this population, without prior investigations.

#### 4.2.2 Medication for obstructive airways

We found that all the registered drugs in asthma treatment were prescribed as fixed dose by age. Lower therapeutic responsiveness has been shown in overweight vs nonobese children; in one study by measuring β‐agonist units dispensed per year and oral corticosteroid dispensing,^24^ and in other studies as response to inhaled corticosteroid.^25^, ^26^, ^27^

#### 4.2.3 Antiepileptic drugs

We found one and four prescriptions of diazepam and lamotrigine, respectively. Both antiepileptic drugs are widely used in pediatric patients. Diazepam is a highly lipophilic drug, which has been found to have an increased *V*_d_ in obese adult patients.^28^ As *V*_d_ is the primary PK parameter of importance when calculating loading dose, the initial dose may have to be increased in overweight/obese children. Opposite, lamotrigine is used for maintenance treatment of epilepsy. CL is therefore the primary PK parameter of importance when dosing. However, the effect of obesity on lamotrigine PK has not been investigated.

The lack of sufficient data on how to dose children who are overweight and or obese in acute care, such as antibiotics, anti‐epileptics, and drugs with narrow therapeutic interval, that is, gentamicin, paracetamol, and diazapam is concerning.

In the light of the prevalence of overweight/obese, especially in children has increased over the last several decades. At present, approximately 31.8% of US children aged 2‐19 years are considered overweight or obese.^29^ Among European countries, overweight/obese in children below the age of ten ranges from more than 40% in southern Europe to less than 10% in northern Europe.^29^ In Denmark, the combined prevalence of childhood overweight/obese is 10%‐12%, in preschool children,^30^ increasing to 15%‐22% during adolescence.^31^ Given prevalence is based on age‐ and gender‐specific BMI. However, definitions of overweight/obese and the reference populations differ between studies and countries.^29^

### 4.3 Main therapeutic classes

In accordance with previous epidemiological studies, we found a high use of respiratory drugs, for example, inhaled steroids and short‐acting beta‐2 agonists 3 and drugs acting on the nervous system. In group N, we found that both methylphenidate and antipsychotics such as quetiapine and aripiprazole were highly prevalent. Of notice, the use of antipsychotic agents is considered very problematic in this population, due to side effects such as weight gain and metabolic syndrome.^32^, ^33^

### 4.4 Using metrics of body size in children with overweight/obesity

Although we found only one prescription had followed such a metric, individualized dosing by metrics of body size may be more feasible in overweight/obese children than common pediatric dosing strategies by TBW or fixed dose by age.^5^, ^6^, ^7^, ^8^, ^9^, ^10^, ^16^, ^17^, ^19^, ^20^, ^23^, ^34^, ^35^, ^36^, ^37^, ^38^, ^38^, ^39^, ^40^, ^41^, ^42^, ^43^, ^44^, ^45^, ^46^ A reflection of the findings in this study, therefore, is that metrics of body size does not seem to be implemented in the clinic unless they have been “transformed” into clinical usefully guidelines. Consequently, there is a risk of supra‐ or subtherapeutic doses. Alazmi et al. estimated inappropriate drug dose prescriptions in 66% of the overweight/obese children, in an outpatient pediatric clinic.^47^ However, general pediatric dosing guidelines, and maximum adult doses were used as references, which is a limitation of the study. Without studies of the individual drug in children who are overweight/obese, it is not possible to decide, which dosing regimens are actually appropriate. In line with this, the Pediatric Pharmacy Advocacy Group recently came with a position statement recommending that weight‐based dosing should be used in patients ages <18 years who are <40 kg and weight‐based dosing should be used in patients ≥40 kg, unless the recommended adult dose for the specific indication is exceed.^48^ In this study, we found that the capping dose was common practice, when the dose exceeded the maximum recommended adult dose. This strategy seems pragmatic, but relies, however, on a precautionary approach rather than evidence, and may not always be applicable. For example, carbamazepine, phenytoin, digoxin, and propofol should be dosed more frequently and in higher dose per kg body weight in normal weight children than in adults.^17^ On the other hand, there exists a well‐known nonlinear relationship between bodyweight and physiological factors such as clearance (CL). Thus, providing maximum adult dose even though limited, may result in supratherapeutic doses.^5^

### 4.5 Strengths and limitations

A major strength of this study is that through manually review of patient records, we were able to define the chosen dosage strategy for each prescription, and included a long‐term period of follow up.

Some obvious limitations are the retrospective design and the method of chart audit with missing data and an underestimation of drug prescription by GPs during follow up. Further, the pharmacodynamics (PD) was not investigated in this study, and due to the retrospective nature of the study, we did not have valid or even the necessary measurements ie visual analog scores (VAS) in accordance to measure effects of analgesics.

## 5 CONCLUSION

This study highlights the shortage of dosing guidelines in children, who are overweight/obese. We found a large interindividual variability in dosing regimens, even in drugs with narrow therapeutic intervals. The clinicians rely on “best practice”, as evidence‐based dosage regimens are missing for many drugs prescribed during childhood.

### 5.1 Perspective

Especially prospective, PK/PD and randomized studies of different dosage strategies are needed to provide information on optimal dosing in children, who are overweight/obese. Meanwhile we encourage the associations of pediatricians in collaboration with clinical pharmacologists and other health care professionals, for example, pediatric pharmacists to standardize dosage guidelines. Individualized dosing strategies and PKPD modeling may be useful tools in this population.

## DISCLOSURES

None declared.
